# Interaction study of *Pasteurella multocida* with culturable aerobic bacteria isolated from porcine respiratory tracts using coculture in conditioned media

**DOI:** 10.1186/s12866-020-02071-4

**Published:** 2021-01-09

**Authors:** Nonzee Hanchanachai, Pramote Chumnanpuen, Teerasak E-kobon

**Affiliations:** 1 Interdisciplinary Graduate Program in Bioscience, Faculty of Science, Kasetsart University, Bangkok, 10900 Thailand; 2 Computational Biomodelling Laboratory for Agricultural Science and Technology, Kasetsart University, Bangkok, 10900 Thailand; 3 Department of Zoology, Faculty of Science, Kasetsart University, Bangkok, 10900 Thailand; 4 Department of Genetics, Faculty of Science, Kasetsart University, Bangkok, 10900 Thailand; 5 Omics Center for Agriculture, Bioresources, Food, and Health, Kasetsart University (OmiKU), Bangkok, 10900 Thailand

**Keywords:** *Pasteurella multocida*, Bacteria-bacteria interaction, Coculture, Porcine respiratory tract, Conditioned media

## Abstract

**Background:**

The porcine respiratory tract harbours multiple microorganisms, and the interactions between these organisms could be associated with animal health status. *Pasteurella multocida* is a culturable facultative anaerobic bacterium isolated from healthy and diseased porcine respiratory tracts. The interaction between *P. multocida* and other aerobic commensal bacteria in the porcine respiratory tract is not well understood. This study aimed to determine the interactions between porcine *P. multocida* capsular serotype A and D strains and other culturable aerobic bacteria isolated from porcine respiratory tracts using a coculture assay in conditioned media followed by calculation of the growth rates and interaction parameters.

**Results:**

One hundred and sixteen bacterial samples were isolated from five porcine respiratory tracts, and 93 isolates were identified and phylogenetically classified into fourteen genera based on 16S rRNA sequences. Thirteen isolates from Gram-negative bacterial genera and two isolates from the Gram-positive bacterial genus were selected for coculture with *P. multocida*. From 17 × 17 (289) interaction pairs, the majority of 220 pairs had negative interactions indicating competition for nutrients and space, while 17 pairs were identified as mild cooperative or positive interactions indicating their coexistence. All conditioned media, except those of *Acinetobacter*, could inhibit *P. multocida* growth. Conversely, the conditioned media of *P. multocida* also inhibited the growth of nine isolates plus themselves.

**Conclusion:**

Negative interaction was the major interactions among the coculture of these 15 representative isolates and the coculture with *P. multocida*. The conditioned media in this study might be further analysed to identify critical molecules and examined by the in vivo experiments. The study proposed the possibility of using these molecules in conditioned media to control *P. multocida* growth.

**Supplementary Information:**

The online version contains supplementary material available at 10.1186/s12866-020-02071-4.

## Background

The porcine respiratory system is exposed to external environments and foreign particles, including bacteria, viruses and pollutants, through inhalation and exhalation processes [1, 2]. Respiratory diseases are associated with economic loss in the swine industry [3, 4]. Metagenomic studies revealed that several bacteria predominantly colonized the porcine respiratory tract, including those in the phyla Firmicutes, Proteobacteria, and Bacteroidetes, and changes in these bacteria were associated with porcine health status [5–8]. Piglets with Glässer’s disease in Spain had a higher number of Proteobacteria in the families *Pasteurellaceae* and *Moraxellacea* and lower number of Firmicutes in the *Ruminococcaceae* family in the nasal cavity compared to the healthy piglets [5, 7]. Huang et al. [8] used 16S rRNA metagenomic sequencing to examine 20 swine lungs. They found that the healthy lungs prevalently had bacteria from the genera *Methylotenera*, *Prevotella*, *Sphingobium*, and *Lactobacillus*, whereas the genera *Mycoplasma*, *Ureaplasma*, *Sphingobium*, *Haemophilus*, and *Phyllobacterium* were abundant in the severe-lesion lungs. The microbial diversity inside these lesion lungs decreased when the population of certain bacteria increased. These studies have raised questions on how these porcine respiratory-tract bacteria interact and control the balance of commensal and pathogenic bacteria in the community.

A member of the *Pasteurellaceae* family, *Pasteurella multocida*, commonly inhabits the nasopharynx of birds and mammals and can be associated with economically significant diseases in pigs, including progressive atrophic rhinitis (PAR) caused by the toxin-producing strains, pneumonic pasteurellosis, and porcine respiratory disease complex (PRDC) caused by multiple pathogens [9–11]. The porcine strains of *P. multocida* commonly belong to capsular types A and D, which could be isolated from the nose, tonsils and upper respiratory tract of both healthy and diseased pigs [12–15]. The non-toxigenic capsular type A strains could be the primary agent of pneumonia and septicemia in 100-day-old pigs [12, 16] and caused dermatitis and nephropathy syndrome (PNDS) in growing and finishing pigs [12, 16]. Several in vitro and in vivo bacteria-bacteria interaction studies focused on the interaction between *P. multocida* and other primary respiratory pathogens in the pathogenic process. The porcine toxigenic capsular type A and D strains of *P. multocida* can be primary pathogens or coinfect piglets with *Bordetella bronchiseptica*, causing PAR under stress and immunocompromised conditions [17]. Colonization of these strains in porcine tracheal rings increased during co-infection with *B. bronchiseptica* [18]. The adherence study of *P. multocida* and *B. bronchiseptica* to swine nasal epithelial cells found that *P. multocida* could not colonize the swine nasal mucosa well compared to *B. bronchiseptica* [19, 20]. The number of *B. bronchiseptica* cells adhered to the nasal epithelial cells was three times higher than the number of *P. multocida* cells, suggesting the opportunistic role of *P. multocida* after *B. bronchiseptica* infection [19, 20]. The co-infection of *P. multocida* with other pathogens could enhance disease damage to the hosts; e.g., promote secure attachment of the bacteria to the bovine respiratory syncytial virus-infected cells [21] and increase inflammatory cells in the coinfected lesions of bronchopneumonia pigs [22]. Recently, Bartkiene et al. developed a combination of a plant extract with a probiotic bacterium *Lactobacillus uvarum* that could inhibit *P. multocida* growth in vitro [23]. This finding has led to hypothesize the role of the porcine respiratory tract normal flora on *P. multocida* growth. However, the interactions of *P. multocida* with the commensal bacteria in the porcine respiratory tract are less understood.

In vitro and in vivo coculture assays are frequently employed to study bacterial interactions. Different bacterial species could be directly cocultured together by the planktonic mixed culture [24–26] with or without physical separation [27] and the host cell model [28]. Indirect coculture could be another option by growing one bacterial species in the culture medium used by another species, also called spent or conditioned medium [29]. De Vos et al. [29] examined the polymicrobial interaction of 72 bacterial samples isolated from 23 individuals with urinary tract infections by using a coculture assay in the spent media. The study found that competitive (−/−) and cooperative (+/+) interactions were more common than exploitive interactions (+/−) and that competitive interactions were enriched among individuals. As explained in the above example, bacterial culture in the conditioned media could be easily monitored and expanded to observe responses by comparing to the control culture. As the interaction studies between *P. multocida* and other commensal bacteria in the porcine respiratory tract remain not well understood, this study aimed to initially determine the in vitro interactions between the porcine capsular type A and D strains of *P. multocida* and other culturable aerobic bacteria isolated from porcine respiratory tracts using the coculture assay in conditioned media. Understanding these interactions would benefit further examination of the in vivo bacterial interactions in the porcine respiratory tract. It would assist the process of respiratory disease control in improving porcine health and welfare.

## Results

### Culturable aerobic bacteria from porcine respiratory tracts

**Fig. 1:**
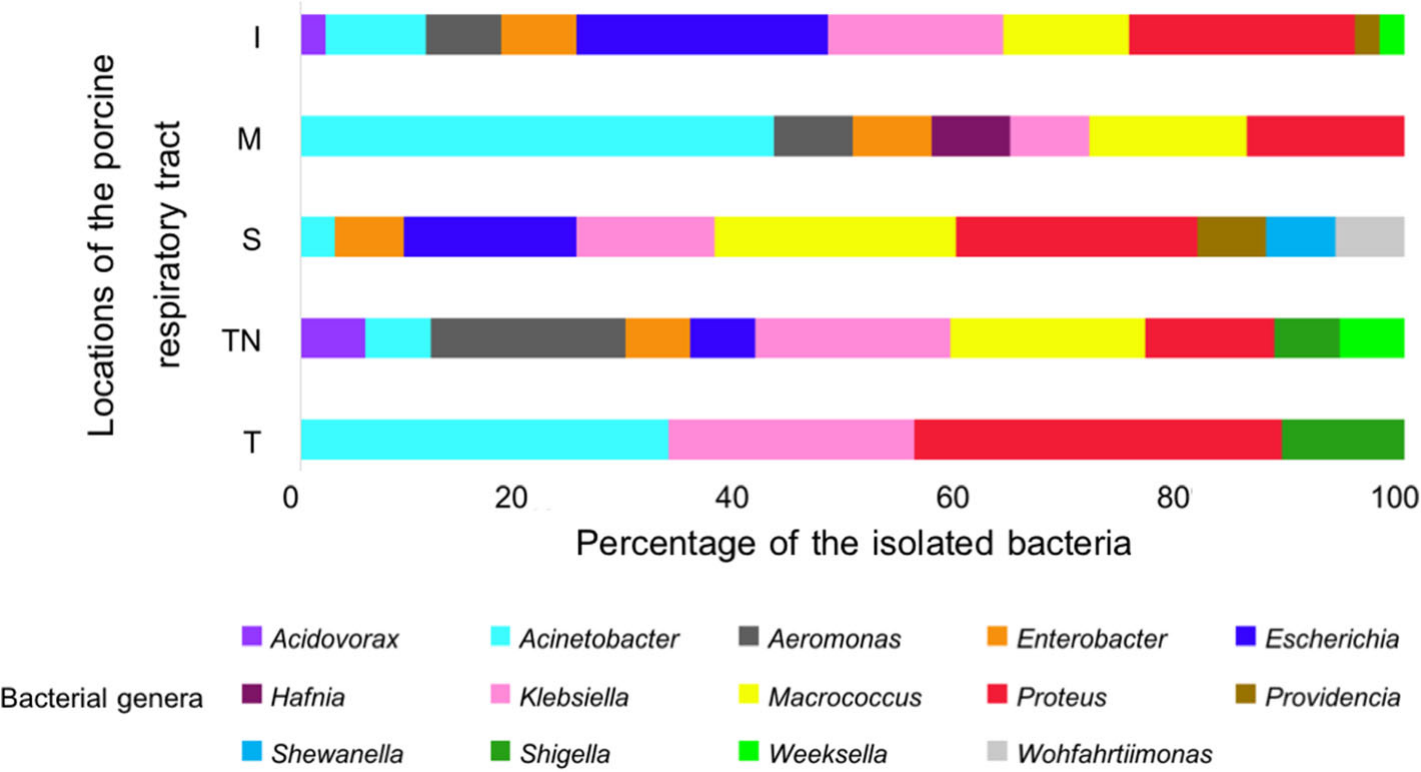
Percentage of isolated aerobic and culturable bacterial genera from different locations of the porcine respiratory tracts. T; Trachea, TN; Tracheobronchial lymph node, S; Apical lobe, M; Cardiac lobe, and I; Diaphragmatic lobe

**Fig. 2:**
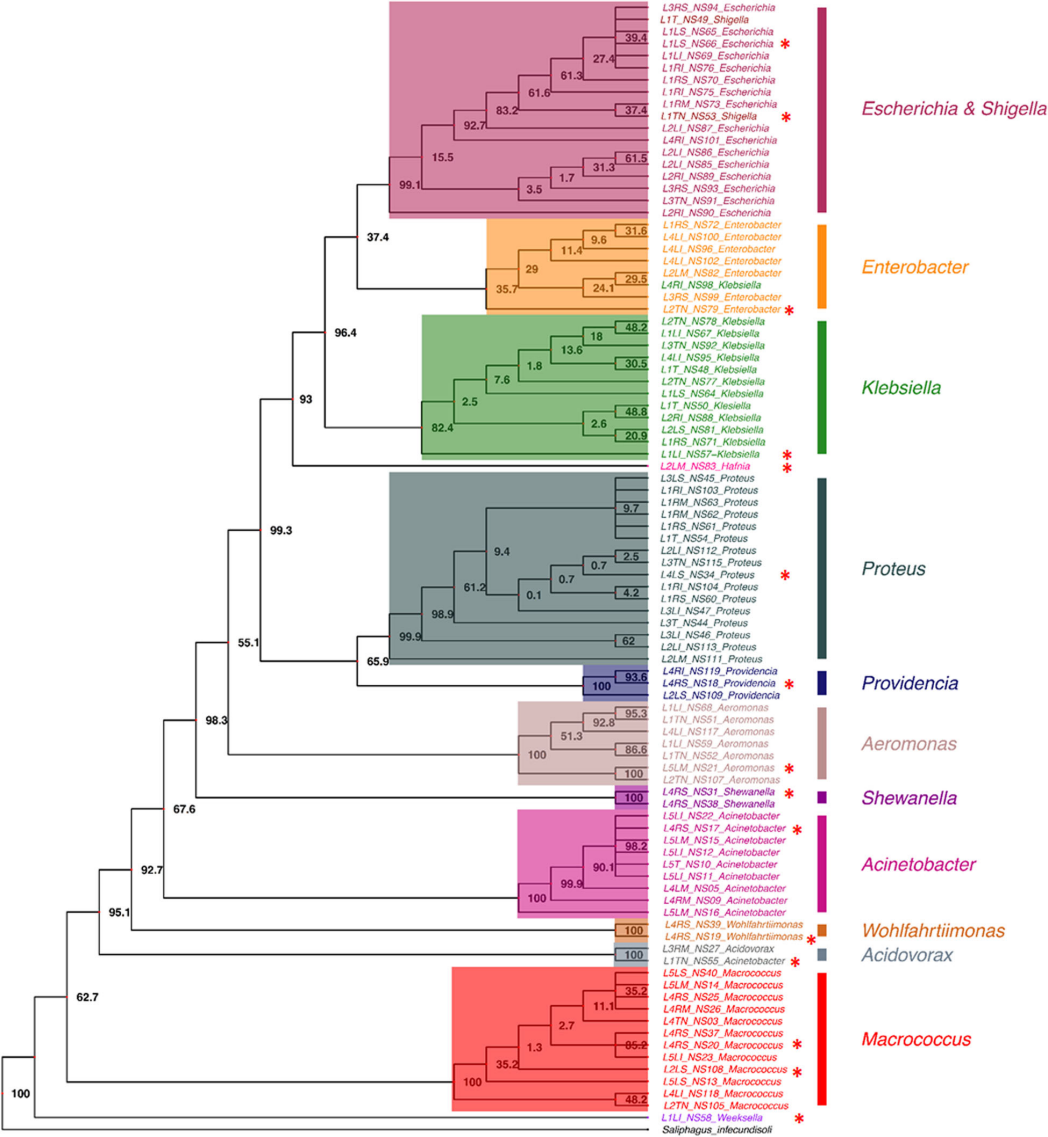
Phylogenetic relationships of the 16S rRNA gene from 93 culturable aerobic bacterial isolates from porcine respiratory tracts constructed by the maximum likelihood model with 1000 bootstraps (shown as percentage numbers at the node of the tree) and using the sequence of *Saliphagus infecundisoli* as an outgroup. Seven major clusters were highlighted with different coloured boxes, and the genera were labelled with different colours on the right of the tree. The red asterisk showed the selected isolates for the coculture assay

One hundred and sixteen aerobic bacterial isolates from five porcine respiratory tracts were successfully cultured from the trachea, tracheobronchial lymph node, apical lobe, cardiac lobe, and diaphragmatic lobe of both the left and right lungs. An average of 23 ± 10 isolates was obtained from each lung. The L1 and L4 lungs had the highest numbers of 31 and 36 isolates, respectively. The majority of the isolates (56%) were from the apical and diaphragmatic lobes of the lungs. Almost 90% of these isolates were gram-negative rod-shaped bacteria that had different colony characteristics, i.e., colony forms (63% circular and 37% irregular), margins (55% undulate, 26% entire, 18% curled, and 1% lobate), and mucosity (66% nonmucoid and 34% mucoid). Ninety-three (80%) of these aerobic culturable bacteria were successfully identified and classified into 14 genera and 21 species (Additional file 1) from seven families of three bacterial phyla (97% Proteobacteria, 17% Firmicutes, and 2% Bacteroidetes), i.e., *Acidovorax*, *Acinetobacter*, *Aeromonas*, *Escherichia*, *Enterobacter*, *Hafnia*, *Klebsiella*, *Macrococcus*, *Proteus*, *Providencia*, *Shewanella*, *Shigella*, *Weeksella*, and *Wohlfahrtiimonas*, based on 16S rRNA sequence analysis (Figs. 1 and 2). The accession numbers of the sequences were shown in Additional file 1. From Fig. 1, the prevalence of the aerobic culturable bacterial isolates in five parts of the porcine respiratory tracts differed. Three genera (*Proteus*, *Acinetobacter*, and *Klebsiella*) were abundant in the trachea (89%). In comparison, ten genera isolated from the tracheobronchial lymph node were the most diverse (53%) with three abundant genera (*Aeromonas*, *Klebsiella*, and *Macrococcus*). Moreover, the three lobes of the porcine lung also showed different abundance: *Acinetobacter* (43%) in the cardiac lobe, *Escherichia* and *Proteus* (43%) in the diaphragmatic lobe, and *Macrococcus* and *Proteus* (44%) in the apical lobe. Phylogenetic analysis of the 16S rRNA sequences clustered these 93 isolates into seven major groups (Fig. 2). The first four groups (60%) were members of the family *Enterobacteriaceae*, including *Escherichia*, *Shigella*, *Enterobacter*, *Klebsiella*, *Hafnia*, *Proteus*, and a small cluster of *Providencia*. The fifth group contained *Macrococcus*, which was the only gram-positive bacterial genus belonging to the family *Staphylococcaceae*. The sixth and seventh groups consisted of the genera *Acinetobacter* (family *Moraxellaceae*) and *Aeromonas* (family *Aeromonadaceae*). The remaining isolates had only one or two members, including *Acidovorax* (family *Comamonadaceae*), *Shewanella* (family *Shewanellaceae*), *Wohlfahrtiimonas* (unclassified bacteria in the class Gammaproteobacteria), and *Weeksella* (family *Flavobacteriaceae*). One isolate was selected to represent each identified genus, except two isolates of *Macrococcus*, NS20 (G5) and NS108 (G7), for the only gram-positive bacterial group as displayed in Fig. 2.

### Growth of the selected aerobic bacterial isolates from the porcine respiratory tracts in different conditioned media

**Fig. 3:**
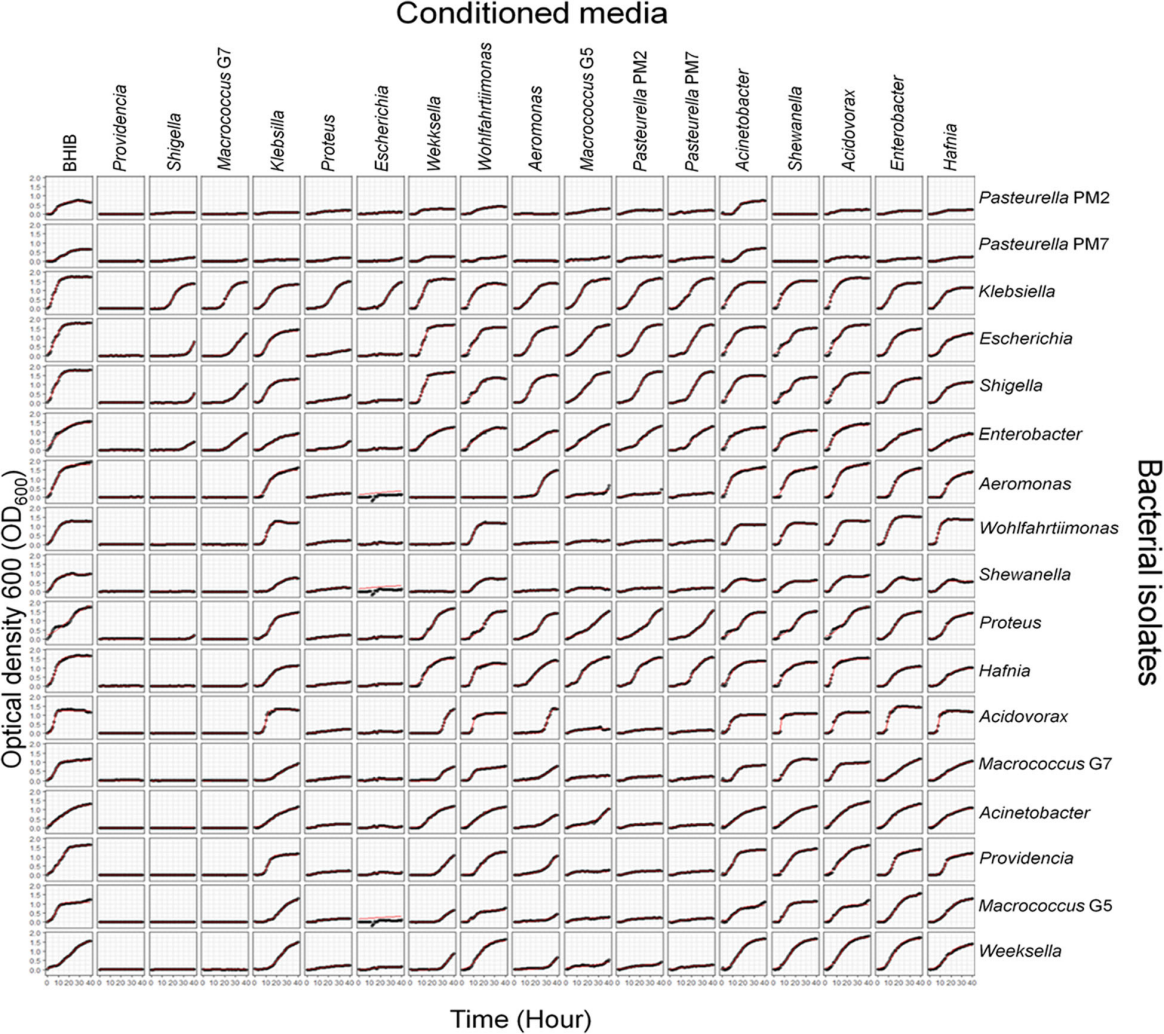
Comparative growth rate of 15 isolates (14 genera) of aerobic bacteria from the porcine respiratory tract and two porcine strains (PM2 and PM7) of *P. multocida* grown in different conditioned media compared to growth in complete medium (BHIB). The optical density at 600 nm was measured hourly for 40 h, and the growth rate was calculated using the logistic equation

Fifteen isolates from 14 genera of the isolated aerobic bacteria from the porcine respiratory tracts and two porcine strains of *P. multocida* with capsular types A and D (PM7 and PM2) were cocultured in the conditioned media (spent BHIB) and the unconditioned media (fresh BHIB), resulting in 289 interacting pairs (17 × 17) as shown in Fig. 3. Nearly all conditioned media could inhibit the growth of these two *P. multocida* strains (the first two rows of Fig. 3), except that of *Acinetobacter*. The conditioned medium of *Acinetobacter* supported or slightly slowed the growth of all tested bacteria. The conditioned medium of *Providencia* inhibited the growth of every isolate, including itself. The media of *Shigella* and *Macrococcus* NS108 (G7) had a lower inhibitory effect on *Klebsiella*, *Escherichia*, *Shigella*, and *Enterobacter*. Conditioned media from *Proteus* and *Escherichia* only supported the growth of *Klebsiella* with a prolonged lag phase. The media of five bacterial samples (*Klebsiella*, *Shewanella*, *Acidovorax*, *Enterobacter*, and *Hafnia*) only inhibited the growth of *P. multocida*. The media of both *P. multocida* strains similarly inhibited *Aeromonas*, *Wohlfahrtiimonas*, *Shewanella*, *Acidovorax*, *Macrococcus*, *Acinetobacter*, *Providencia*, and *Weeksella* as well as themselves. The media of the remaining four samples (*Weeksella*, *Wohlfahrtiimonas*, *Aeromonas*, and *Macrococcus* G5) had different effects on the tested bacteria. Some conditioned media could promote bacterial growth compared to the control. For example, *Weeksella* grew better in the conditioned media of five bacterial samples (*Acinetobacter*, *Wohlfahrtiimonas*, *Shewanella*, *Acidovorax*, and *Enterobacter*)*.*

### Interaction between the porcine strains of *Pasteurella multocida* and the selected aerobic bacteria from the porcine respiratory tracts

**Fig. 4:**
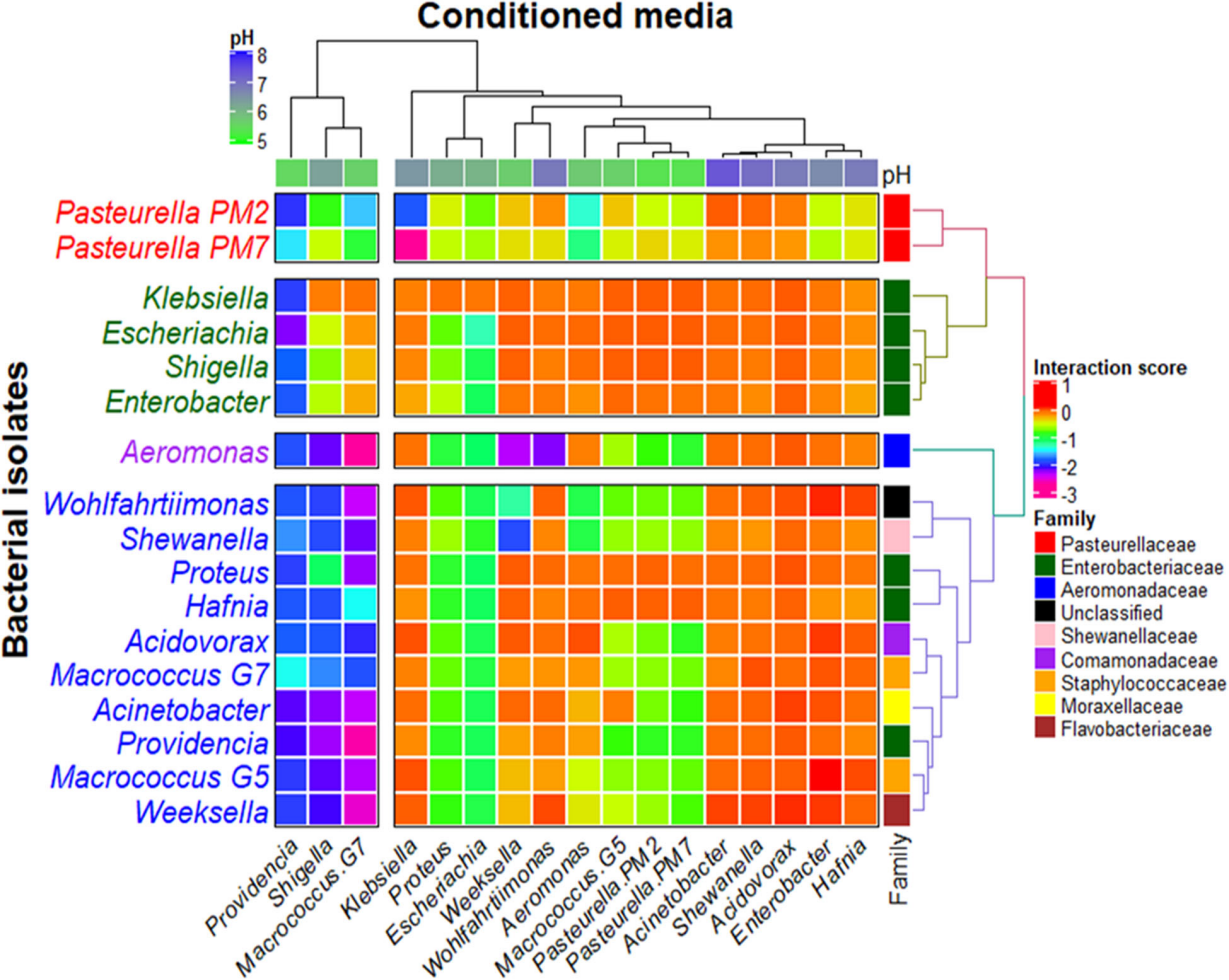
Interactions between 15 selected bacterial isolates from 14 genera with two porcine strains (PM2 and PM) of *P. multocida*. The interactions were examined by a coculturing assay in conditioned media. The interaction score (*ε*) represented the interaction between bacterial isolates: a positive score for a positive interaction and a negative score for a negative interaction. The interaction scores were clustered and labelled by the pH value of the media (horizontal rows) and bacterial family (vertical columns)

This study measured bacterial interactions using the interaction parameter *ε*, which was calculated from the log ratio of maximum growth yield in the conditioned medium compared with that in the unconditioned medium. Pairwise interactions between 17 bacterial isolates revealed that most of the interactions (220 interactions) were negative interactions (*ε* < 0 and the interaction scores of the colour scale between orange and pink in Fig. 4). All negative interactions (−/−) were observed when growing the isolates in the conditioned media from *Escherichia*, *Macrococcus*, *Pasteurella*, *Proteus*, *Providencia*, *Shigella*, and *Weeksella*. Strong negative interactions (59 interactions, *ε* < − 1) were observed in the conditioned media of *Providencia* (17 interactions), *Macrococcus* G5 (12 interactions), *Escherichia* (11 interactions), *Shigella* (11 interactions), and *Weeksella* (3 interactions), and four interactions were observed in the media of *Aeromonas*, *Klebsiella*, and *Wohlfahrtiimonas*. All spent media had a pH between 5.0–7.3, which was lower than the pH of the reference medium BHIB (7.4) (top dendrogram in Fig. 4). Conditioned media from four bacteria (*Aeromonas*, *Klebsiella*, *Macrococcus* G5, and *Providencia*) had a strong negative effect (*ε* < − 1) on *P. multocida* growth and the medium of *Klebsiella* showed the most substantial impact (*ε* = − 1.8 and − 2.6). Notably, the medium of *Providencia* (pH 5.5) had a strong negative interaction with all tested isolates, including itself. The low pH (5.4) of the media from the two *P. multocida* strains resulted in mild to moderate negative interactions with the other tested bacteria. The interaction patterns of *P. multocida* with these 17 conditioned media were separated from those of other bacterial samples (as shown in the right dendrogram) similar to the second cluster of four isolates from the Enterobacteriaceae family and the third cluster of ten samples. Seventeen mild positive interactions (+/+, 0 < *ε* < 0.1) were observed with the media of *Acidovorax*, *Acinetobacter*, *Aeromonas*, *Enterobacter*, *Hafnia*, *Klebsiella*, *Shewanella*, and *Wohlfahrtiimonas*. Six of these interactions (*Acidovorax*, *Acinetobacter*, *Enterobacter*, *Hafnia*, *Shewanella*, and *Wohlfahrtiimonas*) observed in spent media with pH values between 6.5–7.3, which were close to the pH of BHIB. *Weeksella* had positive interactions in five conditioned media, *Acidovorax*, *Acinetobacter*, *Enterobacter*, *Shewanella*, and *Wohlfahrtiimonas*, which was the highest number among the media (highlighted in red in Fig. 4). The conditioned medium of *Enterobacter* supported the highest number of positive interactions with five bacteria, which included *Acinetobacter*, *Acidovorax*, *Macrococcus* G7, *Wohlfahrtiimonas*, and *Weeksella*. A high proportion of mild negative interactions (88 interactions, 0 > *ε* > − 0.1) was observed from bacteria grown in nearly all media, except *Providencia* and *Shigella*. These mild positive and negative interactions could be classified as neutral interactions. However, strong positive interactions were not observed in this study.

**Fig. 5:**
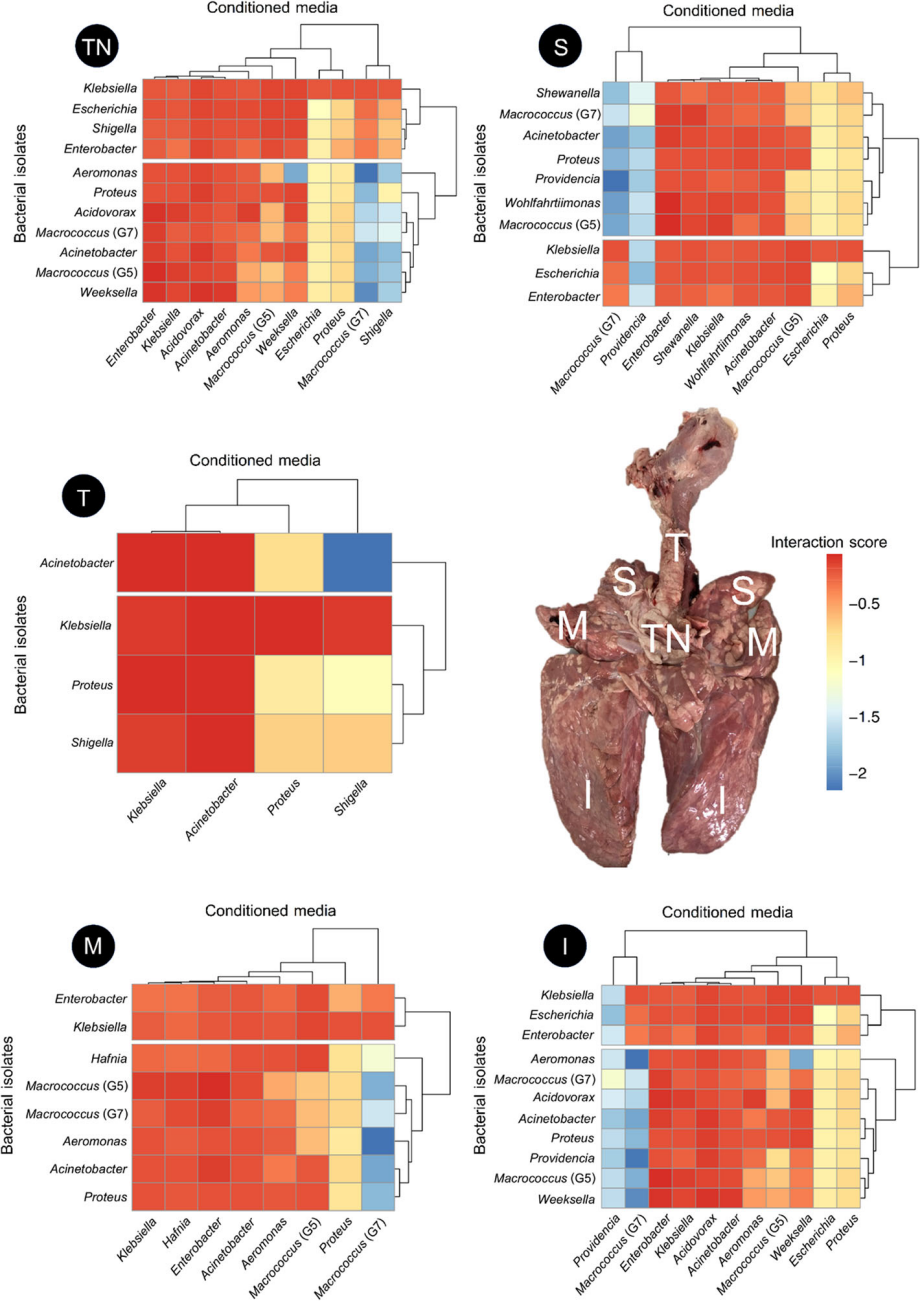
Bacterial interaction values (*ε*) clustered by locations of the porcine respiratory tracts. T, trachea; TN, tracheobronchial lymph node; S, apical lobe; M, cardiac lobe; and I, diaphragmatic lobe of the left and right lungs. A positive interaction score represented a positive interaction (+/+), and a negative interaction score represented a negative interaction (+/−). The top dendrogram shows the conditioned media clustering, and the right dendrogram shows the genera clustering

By comparing the interactions between bacterial isolates found within the same locations of the porcine respiratory tract displayed in Fig. 1, results in Fig. 5 showed that the trachea (T) had the least number of bacterial genera (4 genera) and had *Shigella* as a strong negative influencer. *Macrococcus* G7 and *Shigella* had substantial negative impacts on the others in the tracheobronchial lymph node (TN), while *Acidovorax*, *Acinetobacter*, *Enterobacter*, and *Klebsiella* provided positive support to some bacteria in this group. For the apical, cardiac, and diaphragmatic lobes of the porcine lung, *Macrococcus* G7 was the major bacteria that had a strong negative interaction with the others, except for the apical lobe, in which *Providencia* also exerted a negative effect. These three lobes of the lung shared five common bacteria with mild negative or positive interactions (*Acinetobacter*, *Enterobacter*, *Klebsiella*, *Macrococcus*, and *Proteus*), whereas *Shewanella* and *Wohlfahrtiimonas* were unique to the apical lobe, *Hafnia* was unique to the cardiac lobe, and *Weeksella* was unique to the diaphragmatic lobe.

## Discussion

The porcine respiratory tract has a large mucosal surface area suitable for the colonization of several bacteria, including pathogens [30]. Our study focused on bacterial isolates from porcine respiratory tracts that were culturable and able to grow under aerobic conditions due to the ease of culture and handling so that the isolates could be used for initial coculture experiments to examine their interactions with opportunistic/pathogenic bacteria such as *P. multocida*. From 116 isolates, our study identified 14 bacterial genera, which included four aerobic (*Acidovorax*, *Acinetobacter*, *Weeksella*, and *Wohlfahrtiimonas*) and ten facultative anaerobic (*Aeromonas*, *Escherichia*, *Enterobacter*, *Hafnia*, *Klebsiella*, *Macrococcus*, *Proteus*, *Providencia*, *Shewanella*, and *Shigella*) bacterial genera belonging to seven families (*Aeromonadaceae*, *Comamonadaceae*, *Enterobacteriaceae*, *Flavobacteriaceae*, *Moraxellaceae*, *Shewanellaceae*, and *Staphylococcaceae*) under three phyla (Proteobacteria, Firmicutes, and Bacteroidetes) from the respiratory tracts of healthy pigs. The isolation of Proteobacteria, Firmicutes, and Bacteroidetes could be due to their abundance in the porcine respiratory tract as previously shown from the metagenomic studies of the nasal, oropharyngeal, and lung microbiota [6–8, 31–35]. Some of these 14 bacterial genera were reported in similar locations of the porcine respiratory tract [5–8, 31–40]. *Acinetobacter*, *Aeromonas*, *Escherichia*, *Enterobacter*, *Klebsiella*, *Proteus*, and *Shigella* were found in porcine nasal cavity, oropharynx, trachea, lymph node, and lung [5, 6, 8, 31–37, 40]. However, only *Acinetobacter*, *Klebsiella*, and *Proteus* were isolated from the trachea, lymph node, and lung in the present study (Fig. 1). *Aeromonas* and *Enterobacter* were isolated from lung and lymph node whereas *Shigella* was found in the lymph node and trachea, and *Escherichia* was only found in the lung. The isolation of *Weeksella* and *Shewanella* in the porcine lung agreed with the studies of Correa-Fiz et al. [5], Siqueira et al. [6], and Huang et al. [8]. Our study identified *Providencia* in lung and *Macrococcus* in lymph node and lung consistent with Mann et al. study [32]. Some discrepancies were also observed when compared the present results to other previous studies. Our study additionally found *Hafnia*, *Macrococcus*, *Providencia*, and *Wohlfahrtiimonas* in the porcine lung, *Acidovorax* and *Weeksella* in the lymph node (Additional file 2).

Possible reasons for these discrepancies could be due to different farming environments (area, temperature, and feed) and pig intrinsic factors (genetics, tissue types, age, and sex). For example, comparative nasal microbiota of healthy piglets from farms in the UK and Spain, which had different farm environments shared at least ten bacterial genera [5]. The identification of *Acinetobacter*, *Klebsiella*, and *Weeksella* in our study was consistent with their results, but only *Klebsiella* and *Weeksella* were reported as members of the core nasal microbiota. When compared to the study of oropharyngeal microbiota from healthy piglets in China [7], *Streptococcus* and *Lactobacillus* were the core microbiota in the oropharynx of these healthy piglets consistent with the studies in the UK and Spain [5]. This agreement showed that the nasal and oropharynx of the piglets shared more common bacterial genera compared to the tracheal isolates of the mature pigs in the present study. Our study found five bacteria (*Acinetobacter*, *Enterobacter*, *Klebsiella*, *Macrococcus*, and *Proteus*) to be the core aerobic bacteria inside the lungs, which was also different from a metagenomic study of the microbiota inside the lungs of healthy pigs in Brazil [6]. Their common microbiota included the families *Mycoplasmataceae*, *Bradyrhizobiaceae*, and *Flavobacteriaceae*, whereas only *Aeromonas*, *Escherichia*, and *Weeksella* were shared with the present study. As the nasal cavity, oropharynx, trachea, and lung are connected parts of the porcine respiratory tract for continuous passage of air and exudate, the aerobic and facultative aerobic bacteria could also colonize multiple locations along the tract. For instance, *Acinetobacter* and *Klebsiella* were found in trachea, lung, and lymph node of the present study and in the nasal cavity as reported by Correa-Fiz et al. (2016) [5]. Moreover, the bacterial isolation from three different lobes of the porcine lungs in our study showed some variations in the bacterial genera (Fig. 5), suggesting that the future bacterial sampling of this organ must consider these differences between lobes of the lung. The microbiota of sac and gland-like tissues might be maintained, and the changes may be limited better than the hollow tract with the mucosal surface exposed to the air space within the respiratory tracts. For example, Lowe et al. [35] studied the microbial community inside the tonsils of healthy pigs using culture-dependent and culture-independent methods by sequencing 16S rRNA clone libraries. They were able to identify common bacteria (*Actinobacillus*, *Enterobacter*, *Klebsiella*, *Pasteurella*, *Proteus*, and *Providencia*) from porcine tonsils by both methods, and the results were similar to our work. Although our results covered a subset of the bacterial community in porcine respiratory tracts as previously determined by metagenomics studies, the study successfully narrowed and selected particular groups of aerobically grown bacteria from the diverse community to do the coculture assay and shed light on their interactions with *P. multocida*, which might not be easily assessed by whole-genome shotgun metagenomics.

Opportunistic bacteria, e.g., *Pasteurella*, *Haemophilus*, and *Actinobacillus,* were also abundant in the nasal, oropharynx, and tonsil of healthy pigs but were not isolated under aerobic conditions in this study, which seemed to be a limitation of the bacterial isolation method using the selective media compared to the metagenomics approach which was not required prior bacterial culture [5, 7, 34]. These in vitro-cultured bacterial isolates were good candidates for the coculture assay to examine possible forms of interactions before investigating more complex bacterial interactions in the in vivo or in vivo-like experiments. Most bacterial interactions in this study were negative interactions, which represented the competitive need of these bacteria to share resources and spaces [41]. Certain gram-negative bacterial isolates (*Providencia*, *Shigella*, *Escherichia*, and *Proteus*) in this study yielded conditioned media that strongly inhibited the growth of other bacteria and had similar low pH compared to the fresh BHIB as shown in Fig. 4. These suggested the release of chemicals or the outgrowth of one bacterium, the isolate used to prepared the conditioned medium, would prevent the growth of the other bacteria later inoculated in this medium. The review by Mattingly and Emonet [42] explained complex bacterial chemotaxis behaviours from the growth on the agar plate by the competition between the nutrient-attractive rapid-growing strains and the slow-growing strains which were non-nutrient attractive. The fast-growing ones would replace (competitive exclusion) or dominate (coexisting) by limiting the growth of the low-performing ones. Harrison et al. [43] examined a coculture between siderophore-producing *Staphylococcus aureus* and *Pseudomonas aeruginosa*. These authors found that the absence of free iron induced the production of siderophore by *S. aureus* and increased number of the nonproducing cheater *P. aeruginosa* which would lyse *S. aureus* cells for the irons. Moreover, our coculture assay showed that conditioned media from aerobic bacterial isolates from 13 genera could inhibit the growth of *P. multocida*. The counteract phenomenon had never been reported between *P. multocida* and these 13 bacterial isolates. As previously described in Harrison et al. [43] and Hibbing et al. [41], the conditioned media of these bacterial isolates could lack certain required nutrients for the growth of *P. multocida* such as irons which might be mostly spent during the preparation of the condition media. If *P. multocida* is directly cocultured with these 13 isolates, it might be out-competed and been excluded from the mixture. However, static liquid coculture or biofilm condition could provide multiple niches for these bacteria to compete and perhaps coexist after several generations (Hibbing et al., 2010) [41].

During the preparation of the conditioned media, bacteria multiplied and spent nutrients in the media, so the conditioned media would have fewer nutrients and plenty of metabolites. Normal growth of bacteria in the conditioned media might imply that these bacteria could co-inhabit the same environment (positive and mild negative interactions), while those affected by scarce nutrients, metabolites, and unsuitable pH would not be able to thrive together. The bacterial competition also involves several molecular mechanisms. For example, *Streptomyces* could inhibit antibiotic production by other bacterial competitors to increase its antibiotic production [44]. Barger et al. [45] found that *Streptomyces* secreted a combination of metabolites and enzymes to degrade colonies and cause cellular lysis of *Bacillus subtilis*. Competition could cause disproportional populations in the bacterial community and may alter functional relationships in that ecosystem [46]. These interactions could also change the growth conditions of bacteria in the community, increasing or decreasing community complexity [47]. Aside from the effect of bacterial secretion, the rise of one bacterial population could decrease resource availability for another species in the microbiome system. The results of this study also showed that *P. multocida* had negative interactions with several bacteria and that their conditioned media also inhibited the growth of many bacterial isolates. However, *P. multocida* could not begin its log phase in almost all conditioned media. This pathogen might have to compete and control the growth of the normal flora bacteria to initiate their multiplication. Competition could also occur within the same bacterial population as in the case of *Providencia*, suggesting a process to control the population size and initiate spreading to neighbouring areas. The condition that enhances the growth of the normal flora community would provide an inhibitory effect on the pathogens. Therefore, the conditioned media in this study, particularly those with strong negative effect, have to be further characterized to identify key molecules and mechanisms that could control the *P. multocida* population within the community. In addition to the negative interactions, mild positive interactions were detected for some pairs. Cooperation between microorganisms was reported in a study by Deng and Wang [25], who compared the growth, metabolic activity and enzyme production between pure and mixed cultures in glucose and lignocellulose media. They found that cooperation was common in the lignocellulose media, which promoted positive interactions and synergistic growth. Glucose media promoted negative interactions and competition between organisms in mixed cultures. A study by de Vos et al. [29] also showed that the interaction between bacteria in conditioned media increased bacterial tolerance to antibiotics, and positive interactions were observed under non-antibiotic conditions.

## Conclusions

One hundred and sixteen bacterial isolates were collected from five porcine respiratory tracts, and 93 isolates were phylogenetically classified into fourteen genera based on 16S rRNA sequences. The coculture of 15 representative isolates and two strains of *P. multocida* showed a majority of negative interactions with a few cooperative/positive interactions. All conditioned media, except those of *Acinetobacter*, could inhibit *P. multocida* growth. Conversely, the conditioned media of *P. multocida* also inhibited the growth of eight isolates plus themselves. Thus, this study proposed the possibility of using the molecules in conditioned media to control *P. multocida* growth and further in vivo-like experiments would be examined to understand the inhibitory mechanism better.

## Methods

### Bacterial isolation from porcine respiratory tracts

Five porcine respiratory tracts were collected from slaughterhouses in Nakhon Pathom and Ratchaburi provinces, Thailand, via the assistance of D.V.M. Pichai Joipang from B.F. Feed Co., Ltd. Bacterial samples were isolated from eight different parts of the respiratory tract, i.e., trachea (T), tracheobronchial lymph node (TN), apical lobe (S), cardiac lobe (M) and the diaphragmatic lobe (I) of both the left and right lungs (LXL and LXR, X was the respiratory tract number). Sample sizes and positions were the same for all five respiratory tracts. The samples were spread on tryptose agar supplemented with 5% sheep blood and McConkey agar and then incubated aerobically overnight at 37 °C. Colony morphology was observed, and distinct colonies were selected for further subculture on tryptose blood agar. After incubating aerobically overnight at 37 °C, a single colony was picked and subcultured until the pure isolate was obtained. The pure isolate was smeared on a glass slide and checked for purity and bacterial cell morphology by Gram staining and microscopic observation. Extraction of genomic DNA from the pure isolates was performed using the GF-1 bacterial DNA extraction kit (Vivantis, Malaysia), and the genomic DNA was stored in 50% glycerol with brain and heart infusion broth (BHIB) at − 80 °C until use.

### Bacterial identification by 16S rRNA nucleotide sequencing

Bacterial genera and species were identified by PCR amplification of the 16S rRNA gene using SR–FWD (5′-AGAGTTTGATYMTGGC-3′) and SR–REV (5′-GYTACCTTGTTACGACTT-3′) as forward and reverse primers, respectively [48]. PCR was carried out in a final volume of 20 μL containing 2 μL of DNA template, 0.4 μL of Taq DNA polymerase (Vivantis, Malaysia), 2 μL for each of 2 μM SR–FWD and SR–REV primers, 0.6 μL of 50 mM MgCl_2_, 2 μL of 2 mM dNTPs (Vivantis, Malaysia), 2 μL of 10X Buffer A (Vivantis, Malaysia) and 9 μL of distilled water. PCRs were initially denatured at 95 °C for 5 min, followed by 35 cycles of denaturation at 95 °C for 45 s, annealing at 50 °C for 45 s, extension at 72 °C for 1.35 min, and a final extension at 72 °C for 5 min using a thermal cycler (Bio-Rad Laboratory Inc., Germany). PCR products were quantified and checked for quality using a NanoDrop 2000 (Thermo Scientific, Germany) before separating on 1% agarose gel electrophoresis and visualizing under a UV transilluminator (Bio-Rad, United States). The PCR products were purified using the GF-1 AmbiClean kit (Vivantis, Malaysia) and subjected to Sanger nucleotide sequencing (Macrogen, Korea).

### Selection of representative bacterial isolates by 16S rRNA sequence analysis and phylogenetic reconstruction

The obtained nucleotide sequences of the 16S rRNA gene were trimmed and merged by using the BioEdit program version 7.0.5.3 [49]. The sequences were searched against the NCBI nucleotide database using the blastn program [50] to identify closet bacterial species. The identification was decided based on the Blast query score, e-value equal to 0, and percentage of sequence identity greater than or equal to 99%. As many bacterial isolates were examined and certain isolates belonged to the same genus and species, representative isolates of these bacteria were selected by the following steps. The 16S rDNA sequences of bacterial isolates belonging to the same genus were multiple aligned by using the clustalW algorithm in the BioEdit program version 7.0.5.3 [49, 51]. If the percentage of sequence identity was more than 95%, the sequences were classified into the same genera. If the identity was lower than 95%, the sequences were considered as different genera. The phylogenetic relationship of these 16S rDNA sequences was reconstructed from the aligned sequences based on the maximum likelihood algorithm and Jukes-Cantor substitution model with 1000 bootstrap iterations using the phangorn package in R [52, 53]. The phylogenetic tree was visualized by the ggtree package in R [54]. The phylogenetic data were used to select the bacterial isolates for the coculture assay. At least one isolate representing the same genus was chosen from the cluster and used to prepare conditioned media for the coculture assay.

### Preparation of the conditioned media for the coculture assay

The conditioned medium was spent medium from the culture of a bacterial isolate. All selected bacterial isolates were revived on tryptose blood agar and incubated overnight at 37 °C before subculture into 40 mL of BHIB and incubation for 48 h at 37 °C and 180 rpm. Bacterial cells were pelleted by centrifugation at 4800 x *g* at room temperature for 15 min. The supernatant medium was filtered by a 0.2 μm polyethersulfone (PES) membrane filter (Whatman, United Kingdom) and a 50 mL syringe (Nipro, United States). The pH of all conditioned media was measured using a pH meter (AZ Instrument Corp., Taiwan), and the conditioned media were stored at 4 °C until use.

### Coculture assay and bacterial growth measurement

Two porcine strains of *P. multocida* (capsular types A (PM7) and D (PM2) isolated from pneumonia pigs in Thailand) and the selected isolates of culturable aerobic bacteria were revived on tryptose blood agar and incubated overnight at 37 °C before subculture into 1 mL of BHIB. The coculture assay began by adding 200 μL of the conditioned medium into the nontreated transparent flat-bottom 96-well plate followed by inoculating 0.2 μL of the overnight bacterial culture. Each bacterial isolate was grown in conditioned media from all chosen isolates. The coculture was incubated at 37 °C and 180 rpm for 40 h. The optical density at 600 nm (OD_600_) was measured every hour using a microplate spectrophotometer (PowerWave 340, BioTek, United States), and each condition was performed in triplicate. The bacterial growth rate was calculated using the following logistic equation:

$$ {N}_t=\frac{K}{1+\left(\frac{K-{N}_0}{N_0}\right)\ {e}^{- rt}} $$

where N_t_ represents the population size at time t, and N_0_ is the population size at the beginning of the growth curve. The maximum population size in the particular environment was limited to the carrying capacity parameter K. The OD_600_ values from each condition were fit into this logistic equation to generate the growth curve model by using the SummarizeGrowth function of the Growthcurver package in R [55]. The growth curves were plotted and compared using the ggplot function of the ggplot2 package to visualize the effect of conditioned media on different bacterial isolates [56].

### Determination of the bacterial interactions from the coculture assays

The definition of the bacterial interaction in this study was adjusted from the study of de Vos et al. [29]. The bacterial interaction was expressed as an interaction parameter *ε*, described in the following equation:

$$ \varepsilon =\log \left({N}_c/{N}_u\right) $$

where *N*_*c*_ is the growth yield in the conditioned medium, and *N*_*u*_ is the growth yield in the reference medium or fresh BHIB. The growth yield was defined by the average of the four highest OD_600_ values from the growth curve. The mean of the triplicate maximum growth values in each condition was used to calculate the interaction parameter. A positive *ε* value (*ε* > 0) means that the growth yield in the conditioned medium was higher than that of the reference, indicating the positive or cooperative interaction (+/+) of the two bacterial isolates. A negative or competitive interaction (−/−) corresponds to a negative *ε* value (*ε* < 0). The *ε* ≤ − 1 represents strong negative interaction, whereas the − 1 < *ε* ≤ − 0.1 represents moderate negative interaction and − 0.1 < *ε* < 0 for mild negative interaction. Similarly, *ε* ≥ 1 shows strong positive interaction, whereas the 1 > *ε* ≥ 0.1 shows moderate positive interaction and 0.1 > *ε* > 0 for mild positive interaction. The mild negative and positive interactions would be considered as neutral interaction. These parameters were then used to explain the interactions between *P. multocida* and the selected bacterial isolates.

## Supplementary Information

**Additional file 1.** Details of bacterial isolates. Characteristics and identification of 116 bacterial isolates obtained from the porcine respiratory tracts. Bacterial identification was done by 16S rRNA seqeunce analysis.

**Additional file 2.** Comparison with other published studies. Comparison of bacterial genera isolated from the porcine respiratory tract in other published studies compared to our study.

## Data Availability

All data generated or analysed during this study are included in this published article and its supplementary information files.
