# Supplementary material for: Interaction study of Pasteurella multocida with culturable aerobic bacteria isolated from porcine respiratory tracts using coculture in conditioned media
Source: BMC Microbiol. 2021 Jan 9;21:19. doi: 10.1186/s12866-020-02071-4 (PMC7796573; doi:10.1186/s12866-020-02071-4)
Supplement: Supplementary file 2 — Additional file 2. Comparison with other published studies. Comparison of bacterial genera isolated from the porcine respiratory tract in other published studies compared to our study. [file 12866_2020_2071_MOESM2_ESM.docx]

**Additional table 2** Comparison of bacterial genera isolated from the porcine respiratory tract in other published studies (the first tick) compared to our study (the second tick).

| Bacterial genus | Porcine respiratory region | | | Reference |
| --- | --- | --- | --- | --- |
|  | Lung | Lymph node and tonsil | Nasal, oropharynx, trachea |  |
| *Acidovorax* | ✓^[1, 2]^, ✓ | ✕, ✓ | ✕, ✕ | Huang et al., 2019 [2]  Siqueira et al., 2017 [1] |
| *Acinetobacter* | ✓^[1-3]^, ✓ | ✓^[3-6]^, ✓ | ✓^[7, 8]^, ✓ | Correa-Fiz et al., 2016 [8]  Dosen et al., 2007 [3]  Huang et al., 2019 [2]  Lowe et al., 2011 [6]  Mann et al., 2015 [4, 5]  Siqueira et al., 2017 [1]  Weese et al., 2014 [7] |
| *Aeromonas* | ✓^[10, 11]^, ✓ | ✓^[10]^, ✓ | ✓^[9, 11]^, ✕ | Risco et al., 2013 [11]  Slifierz et al., 2015 [9]  Zhao et al., 2020 [10] |
| *Enterobacter* | ✓^[1, 10]^, ✓ | ✓^[4-6, 10]^, ✓ | ✓^[7]^, ✕ | Lowe et al., 2011 [6]  Mann et al., 2015 [4, 5]  Siqueira et al., 2017 [1]  Weese et al., 2014 [7]  Zhao et al., 2020 [10] |

**Additional table 2** (Continued)

| Bacterial genus | Porcine respiratory region | | | Reference |
| --- | --- | --- | --- | --- |
|  | Lung | Lymph node and tonsil | Nasal, oropharynx, trachea |  |
| *Escherichia* | ✓^[1-3, 10, 12]^, ✓ | ✓^[3-5, 13, 14]^, ✓ | ✓^[7, 10]^, ✕ | Dosen et al., 2007 [3]  Huang et al., 2019 [2]  Lowe et al., 2012 [13]  Mann et al., 2015 [4, 5]  Siqueira et al., 2017 [1]  Stoltz et al., 2010 [12]  Wang et al., 2018 [14]  Weese et al., 2014 [7]  Zhao et al., 2020 [10] |
| *Hafnia* | ✕, ✓ | ✕, ✕ | ✓^[7]^, ✕ | Weese et al., 2014 [7] |
| *Klebsiella* | ✓^[1, 3, 10, 12]^, ✓ | ✓^[6]^, ✓ | ✓^[8, 10]^, ✓ | Correa-Fiz et al., 2016 [8]  Dosen et al., 2007 [3]  Lowe et al., 2011 [6]  Siqueira et al., 2017 [1]  Stoltz et al., 2010 [12]  Zhao et al., 2020 [10] |
| *Macrococcus* | ✕, ✓ | ✓^[4]^, ✓ | ✓^[7]^, ✕ | Mann et al., 2015 [4]  Weese et al., 2014 [7] |

**Additional table 2** (Continued)

| Bacterial genus | Porcine respiratory region | | | Reference |
| --- | --- | --- | --- | --- |
|  | Lung | Lymph node and tonsil | Nasal, oropharynx, trachea |  |
| *Proteus* | ✓^[10]^, ✓ | ✓^[6, 13]^, ✓ | ✓^[7]^, ✓ | Lowe et al., 2011 [6]  Lowe et al., 2012 [13]  Weese et al., 2014 [7]  Zhao et al., 2020 [10] |
| *Providencia* | ✕, ✓ | ✓^[6, 13]^, ✕ | ✓^[7]^, ✕ | Lowe et al., 2011 [6]  Lowe et al., 2012 [13]  Weese et al., 2014 [7] |
| *Shewanella* | ✓^[1, 2]^, ✓ | ✕, ✕ | ✕, ✕ | Huang et al., 2019 [2]  Siqueira et al., 2017 [1] |
| *Shigella* | ✓^[1, 10]^, ✕, | ✓^[10, 13]^, ✓ | ✓^[14]^, ✓ | Lowe et al., 2012 [13]  Siqueira et al., 2017 [1]  Wang et al., 2018 [14]  Zhao et al., 2020 [10] |
| *Weeksella* | ✓^[1]^, ✓ | ✕, ✓ | ✓^[8]^, ✕ | Correa-Fiz et al., 2016 [8]  Siqueira et al., 2017 [1] |
| *Wohlfahrtiimonas* | ✕, ✓ | ✕, ✕ | ✓^[9]^, ✕ | Slifierz et al., 2015 [9] |
